# Comparing analog and digital neurocognitive tests with older adults: a study of the ISPOCD battery vs. a digital test battery from Mindmore

**DOI:** 10.1186/s12877-023-04648-w

**Published:** 2024-01-08

**Authors:** Anahita Amirpour, Jeanette Eckerblad, Lina Bergman, Ulrica Nilsson

**Affiliations:** 1 Division of Nursing, Department of Neurobiology, Care Sciences and Society, Karolinska Institutet, Alfred Nobels Allé 23, C4, 141 83 Stockholm, Sweden; 2 Perioperative Medicine and Intensive Care, Karolinska University Hospital, Stockholm, Sweden

**Keywords:** Neuropsychology, Neurocognitive test batteries, Postoperative cognitive complications, Postoperative neurocognitive disorder

## Abstract

**Background:**

Delayed neurocognitive recovery and neurocognitive disorder are common postoperative complications among older adults. The assessment of these complications traditionally relies on analog neurocognitive tests, predominantly using the test battery from the ISPOCD-study as the standard approach. However, analog tests are time-consuming and necessitate trained staff which poses limitations. The potential availability of a digital neurocognitive test as an alternative to the ISPOCD remains unknown. We conducted a comparative study between the analog test battery from ISPOCD and the self-administrated digital test battery developed by Mindmore.

**Methods:**

We conducted a crossover study with 50 cognitively healthy older adults ≥ 60 years of age recruited in Stockholm Sweden, between February and April 2022. The primary outcome focused on measuring comparability between the two test batteries. Our secondary outcomes included assessing participants’ perceptions and attitudes about the tests with qualitative interviews and their usability experiences.

**Results:**

Fifty older adults, mean age 76, female 56%, with a university or college degree 48% participated in the study. The sub tests in two test batteries demonstrated a medium–large correlation (*r* = 0.3–0.5), except for one measure. For four out of six measures, significant differences were found with medium to large effect sizes, ranging from 0.57–1.43. Two categories were recognized in the qualitative analysis: *self-competing in a safe environment,* and *experience with technology.* Participants expressed feeling safe and at ease during the assessment, with some preferring the digital test over the analog. Participants reported a high level of usability with the digital test and a majority participants (*n* = 47) reported they would undergo the digital test for a potential future surgery.

**Conclusions:**

The digital test battery developed by Mindmore offers several advantages, including rapid access to test results, easy comprehension, and use for participants, thereby increased accessibility of cognitive screening.

**Trial registration number:**

NCT05253612; ClinicalTrials.gov, 24/02/2022.

## Background

Worldwide, by 2030, one in every six persons will be above the age of 60 [1], and in 2050, the worldwide average lifespan will be about 77.2 years [2]. This growth in the aging population and the increase of cognitive disorders will have a significant impact on anesthesia and surgical practice, since more than 40% of all surgeries in the Western countries are performed on adults over 65 years of age [3].

Delayed neurocognitive recovery (dNCR) and postoperative neurocognitive disorder (p-NCD), previously known as postoperative cognitive dysfunction (POCD) [3, 4], arises from surgical stress [3] and represent two of the most common postoperative complications among older adults [5]. Delayed neurocognitive recovery typically manifests within the first month after surgery while p-NCD can persist between 1–12 months [3, 5, 6]. Both conditions impact multiple cognitive domains including attention, executive functions, processing speed, and memory [7]. Moreover, they are related to loss of independence [3, 8], increased risk of falls, prolonged hospital stay, increased health care costs [6], increased risk of developing dementia [6], and increased risk of mortality [9].

Factors such as multimorbidities or frailty significantly affect postoperative recovery [3] in older adults, making them more susceptible to experiencing dNCR or p-NCD [6]. Preexisting cognitive impairments strongly correlates with the development of cognitive complications after surgery [10, 11]. Unlike acute postoperative delirium, dNCR and p-NCD do not constitute clinical diagnoses [5] and cognitive performance is rarely assessed with objective neurocognitive tests in high-risk patients like older persons. Furthermore, a substantial part of postoperative recovery occurs after hospital discharge [3, 12] potentially allowing neurocognitive disturbances to go unnoticed by healthcare professionals.

### Neurocognitive tests

Neurocognitive tests are required to detect impairments in cognitive function, such as problem solving, processing speed, executive functions, attention, mental flexibility, and memory [13]. The analog test battery (conducted with pen-and-paper and test leader) from the well-known International Study of Postoperative Cognitive Dysfunction (ISPOCD) [14] measures several cognitive domains (Table 1) and has been used in the perioperative setting over the past two decades [15–17]. The ISPOCD analog test battery is based upon traditional tests such as the Rey Auditory Visual Learning Test (RAVLT) [14]. In a recent systematic review, around 86% of neurocognitive tests included in previous perioperative research are analog [7]. However, analog tests are time-consuming, increase the workload of healthcare professionals, require specially trained staff and are at risk of administration bias [18, 19]. Digital tests deliver rapid screening of cognitive impairments, and can be self-administered with validated scores and are therefore easily used on a large scale in clinical settings [20]. Digitalization increases the accessibility of cognitive screening since it can be performed at the patient’s home and other living facilities [19]. Moreover, digital neurocognitive tests minimize administrative burden for health care professionals [21], and provide better diagnostic performance than analog ones [19]. To our knowledge, there is no digital version of the ISPOCD test battery available and a few earlier studies have used digital and analog cognitive tests in the surgical population [22–25], yet the choice of cognitive tests varies and none of them has been compared to the ISPOCD test battery [14].

**Table 1:** Included tests from the International Study Group of Postoperative Cognitive Dysfunction (ISPOCD) and Mindmore batteries

Cognitive Areas	Analog test battery from the ISPOCD	Digital test battery from Mindmore	Variations
Verbal episodic memory	Visual Verbal Learning Test (VVLT) includes a delayed recall	Consortium to Establish a Registry for Alzheimer’s Disease (CERAD), including delayed recall and recognition trial	**VLT: 15 words × 3 trials** **CERAD: 10 words × 3 trials**
Executive, visuospatial	Concept Shifting Task (CST)	Trail Making Test (TMT- A & B)	CST: 16 circles × 3 TMT: 25 circles × 2
Executive, selective attention	Stroop Colour-Word test (SCW)	Stroop Colour-Word Test (SCW)	Analog Stroop 40 words × 3 Digital Stroop: 24 words × 1
Executive, attention, tempo	Letter Digit Coding Test (LDC)	Symbol Digits Processing Test (SDPT)	LDC: 60 s SDPT: 90 s

The Mindmore test battery is a self-administered collection of traditional cognitive tests that have been digitized and provided on a tablet for the purpose of assessing cognitive functioning in patients suspected of cognitive decline [26, 27]. The battery was specifically developed by licensed psychologists and measures a range of cognitive domains [26, 27]. As a result, Mindmore provides a comprehensive assessment of cognitive functioning and is designed to discriminate between normal and non-normal test results [27], making it easier to identify cognitive impairment in patients undergoing surgery. Furthermore, the self-administered nature of Mindmore allows for easy implementation in a perioperative setting, where patients may not have access to trained staff to administer traditional cognitive tests. An equivalence study has been conducted between the Mindmore tests (Rey- Auditory Verbal Learning Test; RAVLT, Corsi, Paced Auditory Serial Addition Test; PASAT, Trail Making Test A + B; TMT A + B, Stroop, Boston Naming Test-15; BNT-15) and the underlying traditional analog tests, demonstrating comparable results between the two and providing evidence of the validity of the Mindmore screening battery [27].

This study’s aim is to evaluate whether the analog test battery from the ISPOCD is comparable to the self-administered digital test battery developed by Mindmore. We also examine the acceptability and usability of the digital test battery on the tablet and explore participants’ perceptions and preferences of the neurocognitive assessment. Consequently, this study contributes to the understanding of neurocognitive assessments in the field of perioperative neurocognitive disorders.

## Methods

### Participants and recruitment

We conducted this randomized crossover study from February–April 2022 at Karolinska Institutet, Stockholm Sweden. We recruited 50 adults from senior organizations, activity centers, and from Karolinska Institutet’s website for study subjects. The sample size estimation was determined based on previous cross-over studies involving older adults undergoing analog and digital neurocognitive tests [28, 29]. The first author provided information about the study to potential study participants and ensured their eligibility. Inclusion criteria for participants were as follows: ≥ 60 years, fluency in Swedish, no alcohol or drug dependencies, no severe psychiatric or neurological illnesses, and no significant uncorrected visual or auditory impairments. Participants self-reported these criteria. At the conclusion of the study, all participants were offered a summary of their digital test results.

### Measurements & procedure

Primary outcome was to measure comparability between the analog test battery from the ISPOCD and the digital test battery from Mindmore. Table 1 displays the analog and digital test batteries.

The test batteries include the following:

- *Verbal episodic memory*
  Visual Verbal Learning Test (VVLT) presents 15 words visually over three trials, with delayed recall after 20 min. Consortium to Establish a Registry for Alzheimer’s Disease (CERAD) word list learning, presents a 10-word verbal learning test with three trials, a recall after approximately 7 min, and a recognition trial.
- *Executive functions and visuospatial*
  Concept Shifting Task (CST) involves 16 circles on paper. In Part A, numbered 1 to 16, the participant draws lines over each number. Part B includes letters from A to P, and Part C pairs digits and letters in ascending order. Trail Making Test A + B (TMT) involves 25 circles in *each part, including drawing lines in ascending order. Part B pairs 13 digits and 12 letters.*
- *Executive functions, attention*
  The Stroop Colour-Word Test (SCW) includes 40 words spelling out a color printed in contrasting ink colors. Participants must state the ink color rather than the word meaning. Mindmore's Stroop Colour-Word Test (MSCW) consists of 24 words spelling out a color, printed in contrasting ink colors. Participants are instructed to identify the printed color rather than the word meaning.
- *Executive functions, visuospatial*
  The Letter Digit Coding Test (LDC) involves matching letters with specific digits for 60 s.
  The Symbol Digits Processing Test (SDPT) randomizes symbols to minimize practice effects. Participants associate a symbol in the center with a digit on a 3 × 3 grid. The final score is the number of correct matches in 90 s.

We selected the test battery from Mindmore (Table 1) in consensus with their research team of psychologists with knowledge of neurocognitive assessments and tailored our choices to match our older adult population.

The analog test battery from the ISPOCD [14] started with the VVLT [30] where the word chain was presented on a laptop. The battery continued with CST, SCW, finalized with LDC [30] conducted with pen and paper. Before initiating the three last tests, the test leader conducted a practice trial. The digital test Mindmore was self-administered on a 12.3-inch touch screen tablet (10.1″ Windows) and consisted of an audial and visual introduction on the tablet before each battery. The battery proceeded in the following order: TMT, CERAD, MSCW, and SDPT. Each test started with a practice trial, except for the CERAD. The practice trial involved providing participants with feedback. However, if a participant made five errors during the practice trial, it was discontinued, while the rest of the battery continued.

The study’s secondary outcome was to measure participants’ perceptions about the two tests and their attitudes about the digital tablet with interviews and usability experiences using a modified version of the System Usability Scale (SUS) [31]. The participants rated in questionnaires how difficult, stressful, and surmountable the tests were on a 5-point scale ranging from 1 (not at all) to 5 (extremely); whether they found the tests to be challenging (yes/no); and what their preferences were (analog/digital). Furthermore, participants were also asked if they would take the digital test to evaluate their cognitive performance following a potential surgery and, if yes, how frequently. We also measured incidence of depressive symptoms using the 15-item geriatric depression scale (GDS-15) [32] since depression is linked to decreased cognitive performance [33], Therefore, the GDS was used to screen for symptoms of depression at the time of testing to ensure that depression did not confound the results. Furthermore, we measured concentration difficulties using one of the items about concentration difficulties (from 0, “none of the time”, to 10, “all of the time”) included in Swedish Quality of Recovery (SwQoR) [34]; see published study protocol for further details [35].

Each participant underwent both test batteries, with two weeks apart [35]. We randomized the cross-over groups using virtual research software [36] and each participant was blinded to which test they would take first, 29 participants started with the analog test and 21 participants started with the digital test. The first author and two research assistants with backgrounds in psychology were trained in the psychometric properties of the tests. We administered the ISPOCD-battery and monitored the self-administered Mindmore battery, to prevent use of smartphones or movement of tablet during assessment. The same test leader oversaw both test sessions for each participant. Both test sessions took place in a quiet room on the premises of the university, with only the test leader and participant present; except for two study participants who took the tests in their private homes with one of the test leaders.

### Statistical analysis

We conducted descriptive analyses for age, gender, and education level; and paired-samples t-tests to compare mean scores of the tests, measure perceived stress, difficulties between the cognitive tests, how surmountable they were perceived to be. Chi-square tests of independence were used to explore the relationship between attitudes about the tests and gender. We compared the analog versus the digital test using the Pearson correlation coefficients (*r)* or Spearman correlation. We applied Cohen’s d for effect size to interpret mean differences between the two test batteries, with cutoff scores of 0.2, 0.5 and 0.8 indicating small, medium, and large effect sizes, respectively [37]. The scores from the SUS are presented as mean SD and not on the original 100-point scale [31]. A two-tailed *p*-value lower than 0.05 was considered statistically significant. We conducted all statistical analyses using IBM SPSS version 28 (IBM Corp., Armonk NY).

### Qualitative outcome

We included twenty participants from the total population (*n* = 50) for a follow up qualitative interview to explore their perceptions of the test sessions and of being assessed for neurocognitive performance. We drew a strategic sample of interviewees to obtain variation based on age, educational background, and gender. The sample size was built on the concept of information power and determined within the data collection process [38]. The second author, who has previous experience with conducting qualitative studies (and who was not involved in data collection), interviewed all participants after both test sessions had concluded.

All interviews were semi-structured and followed an interview guide with questions such as “*how did you feel when you underwent the cognitive tests?*” and *“do you use digital devices?”*. The second author conducted and audio-recorded all interviews on the phone and all interviews were transcribed verbatim and assembled in Microsoft Excel (Microsoft Corporation, Redmond, WA). The first author conducted an inductive content analysis according to Graneheim & Lundman [39] in order to identify the meaning units which were related to the aim of the study, and analyzed and discussed the material with the third author. All authors were involved in the discussion about the meaning units to ensure credibility. We wanted to identify clear patterns across the dataset in order to capture the participant’s situational feelings and attitudes about the cognitive tests on a manifest level (i.e., close to the text), with a low level of interpretation [39]. The qualitative analysis processed as follows: 1) identify meaning units, 2) condense the meaning units without losing context, 3) code, 4) final categorization. The final structure was created after all authors had reviewed the categories and data.

## Results

### Demographics of the sample

The main demographic characteristics of the participants are presented in Table 2. Mean age was 76 years, 56% of participants were female, and 48% had obtained a university degree. Participants had low scores on the GDS (i.e., no depression) and low scores of concentration difficulties on both test sessions (Table 2).

**Table 2:** The main characteristics of the 50 study participants

Characteristics	Total sample (*n* = 50)	Qualitative interview sample (*n* = 20)
Age years, mean SD (range)	76 (64–88)	77 (66–85)
Gender n female/male (%)	28(56)/24 (44)	10/10 (50/50)
**Highest level of completed education n (%)**		
Elementary school	4 (8)	3 (15)
High school	13 (26)	8 (40)
Tertiary education	9 (18)	2 (10)
University or college degree	24 (48)	7 (35)
Geriatric Depression Scale-15 score, 0–15 first session, mean (SD)	1.3 (1.3)	
Geriatric Depression Scale -15 score, 0–15 s session, mean (SD)	1.4 (1.5)	
Concentration difficulties Analog 0–10 mean (SD)	2.2 (2.8)	
Concentration difficulties Digital 0–10 mean (SD)	2.5 (3.0)	

### Comparison between the analog and digital tests

The mean time for the introduction and conducting the analog test with the test leader was 20 min and the mean time for the introduction and self-administering the digital test was 27 min. The relationship between the two tests varied: SCW/MSCW numbers of errors, time for CST part C/TMT B, and scores in VVLT/CERAD showed a medium–large correlation (r/ρ = 0.3–0.5). Notably, the time for SCW (seconds) and MSCW (average milliseconds) had a positive Spearman correlation (ρ = 0.74). In contrast, the correlation for CST/TMT B in terms of the number of errors was low. Paired samples t-test were utilized for comparing mean test scores, and five of six measures were statistically different, *p* < 0.05. The Cohen’s d effect size of mean differences was medium-large (0.57–1.43) in four measures, and small in SCW/MSCW and LDC/SDPT (Table 3).

**Table 3:** Comparison between the analog and digital test batteries

**Tests**	**Correlation**	**Mean (SD) analog**	**Mean (SD)** **Digital**	***p*-value **^**c**^	**Cohen’s d**
VVLT (range 0–45) /CERAD (range 0–30) cumulative	0.47^a^	22.7 (5.2)	18.5 (3.9)	< 0.001	0.86
VVLT (range 0–15) /CERAD (range 0–10) delayed recall, number of words	0.52^a^	8.0 (2.8)	5.8 (1.8)	< 0.001	0.90
CST, time for part C/ TMT B (s)	0.48^a^	38.4 (12.5)	97.2 (45.4)	< 0.001	1.43
CST, number of errors in part C/TMT B error	0.19^a^	1.1 (2.6)	4.7 (6.2)	< 0.001	0.57
SCW/MSCW, number of errors in part 3/ SCW test	0.30^b^	1.2 (2.1)	0.7 (1.4)	0.74	0.25
LDC/SDPT	0.50^a^	27.9 (6.1)	31.0 (7.7)	0.003	0.43

*VLT* Visual Learning Test, *CERAD* Consortium to Establish a Registry for Alzheimer’s disease, *CST* Concept Shifting Task, *TMT A* Trail Making Test part A, *TMT B* Trail Making Test part B, *SCW* Stroop Color Word test, *MSCW* Mindmore Stroop Color word test, *LDC* Letter Digit Coding, *SDPT* Symbol Digits Processing Test

^a^Pearson, *r,* correlation significant at 0.01 level

^b^Spearman *r*_*s*_*,* correlation significant at 0.01 level

^c^Paired-samples t-test between analog and digital tests

### Attitudes about the analog and digital tests

While there was no statistically significant difference between the two types of tests in terms of perceived difficulty, stressfulness, or how surmountable participants experienced them to be, a slightly larger number of participants reported a preference for the digital test (*n* = 28, 56%) compared to the analog test (*n* = 20, 40%), However, this difference did not reach statistical significance (Table 4).

**Table 4:** Respondent attitudes towards both tests

Questionnaire	Range	Analog test	Digital test	*p*-value
Difficult mean (SD)^c^	not at all (1) -extremely (5)	1.9 (0.9)	1.8 (0.9)	0.5^a^
Stressful mean (SD)^c^	not at all (1) -extremely (5)	2.0 (1.0)	2.0 (1.0)	0.7^a^
Surmountable mean (SD)	not at all (1) -extremely (5)	4.0 (0.8)	3.9 (1.0)	0.4^a^
Challenging test (n%)	Yes/No	25 (50)/25(50)	30(60)/20(40)	0.8 /1.0^b^
Your preference^d^(%)		20 (40)	28 (56)	

^a^A paired samples t-test did not reveal significant differences between the tests

^b^A chi square test for independence indicated no significant association between attitudes about challenging test or gender

^c^1 response missing

^d^2 responses missing

Most participants (*n* = 47) reported they would undergo the digital test for a potential future surgery; some preferred the frequency of the assessment to be every day (*n* = 11), every other day (*n* = 8), once a week (*n* = 11), every other week (*n* = 5), once a month (*n* = 4), or another time interval (*n* = 9) where a few expressed “as often as it takes”. Moreover, there was no statistical difference on how challenging the tests were rated between the two genders.

### Usability of the digital test

The usability experience related to the digital test was largely positive (Table 5), the Mindmore tablet was simple to use, uncomplicated and quick to adopt. Furthermore, participants strongly agreed that they felt confident using the tablet and stated that it could be used for repetitive measures without requiring technical assistance.

**Table 5:** Participants’ usability experience of the Mindmore tablet

Modified, shortened SUS	Mean (SD)
	Strongly disagree 1 – 5 Strongly agree
I thought the system was easy to use	4.6 (0.7)
I think it’s possible to use this system for repetitive measures	4.5 (0.7)
I felt confident using this system	4.2 (1.3)
I would imagine that most people would learn to use this system quickly	4.0 (0.9)
I think that I would need the support of a technical person to be able to use this system	1.8 (1.2)
I found the system cumbersome to use	1.6 (1.2)

### Qualitative findings

Twenty of the fifty participants completed qualitative interviews: ten women and ten men, ages 64–85 years, with different educational backgrounds (Table 2). Two categories were identified from the interview material: 1) self-competing in a safe environment, and 2) experience with technology. These categories are presented below with associated quotes.

#### Self-competing in a safe environment

All the interviewed participants expressed an overall pleasant test session experience. The positive experience was multifaceted; being comfortable, feeling safe, and experiencing assurance from the test leader. The test leader fostered a welcoming environment by being neutral, open, and intuitive to the participant. Even though the participants were expected to demonstrate their cognitive capacities during the test, they felt at ease doing so:

“I thought it (the test session) was very calm and sober-minded. And there was only one person (the test leader) there, nothing strange at all.”

Some participants reported that they would sharpen up prior the test and competed against themselves, to demonstrate their own abilities:

“Uhm, I was a bit overly excited by myself, because I wanted to be the best (laughs)…But it wasn’t any negative stress, just excitement.”

However, some participants recalled experiencing pressure and forgetfulness during the test sessions. The pressure was either connected to the participant’s high expectations of themselves or related to the actual execution of the test:

“I got so stressed; I don’t even remember what the color (Stroop color word test) was called.”

Several participants also mentioned how human-to-human interaction made the test experience feel safer:

“From the starting point of my own experiences, I would say it feels kind of safe when there is a living creature beside me, than a…. yeah, something electronic that you’re supposed to handle yourself.”

#### Experience with technology and test preferences

Having prior experience with smart phones and computers was described as a sign of familiarity with digital devices and a willingness to acquire new technical skills:

“Yes, I am used to it (using technology). I sit a lot with the computer, and I try to learn new things too, so you could say that I’m used to it.”

A few participants spoke about having very little experience with digital devices, but still preferred taking the digital test:

“I was quite surprised by myself, because I thought the computer (the tablet) was a lot better, I’m a big opponent of computers…but I thought this was much better. You don’t need to rustle papers all the time”

Several participants expressed they would rather undergo the digital test, since it had straightforward instructions and was easy to learn:

“Yes, maybe it was a bit easier when I did it on my own...yes. It wasn’t like…You did not get performance anxiety when you didn’t know the answer.”

However, a few participants expressed how they had longer and more frequent experiences using analog tools in their lives, and preferred the analog test:

“No, I think the analog test was much better than the digital one. It was easier to understand because someone explained it to you. You can’t ask the tablet; it will not respond.”

## Discussion

In this study, we investigated the comparability of the analog test battery ISPOCD to the self-administered digital test battery Mindmore. Overall, most of the analog tests were moderately comparable and in moderate agreement with their digital counterparts. These findings are in line with other studies comparing digital and analog cognitive tests [19, 27, 40, 41].

In four of six measures the mean test scores were statistically different which is related to the test differences. For example, the VVLT test in the ISPOCD battery consists of 15 words, whereas the CERAD word learning test only includes 10 words. Similarly, the CST part C has 16 alternating circles of numbers and letters, while the TMT B has 25 circles. These differences in item counts may contribute to the observed differences in mean test scores between the two test batteries. Moreover, CST involves crossing out numbers and letters with a pencil, whereas TMT requires drawing lines with the index finger between the circles. There was no correlation between CST part C and TMT B, which corresponds with a study comparing the paper version of the TMT and its digital counterpart [41]. However, several studies have proved that the paper-based TMT and its digital counterpart are comparable [27, 42, 43]. Additionally, the outcome measure for the paper-based version of Stroop was the total time taken to complete the task, whereas for the digitized version, the response time for each word was recorded, and the average response time for correct responses was calculated as the outcome measure. Nonetheless, both tests shared the same objective of assessing the ability to inhibit a response.

The reported high usability of the digital test is promising, which leads us to the next step: assessing the Mindmore tablet in a busy perioperative setting with older adult patients. Usability is one of the key requirements of cognitive screening tools in a recent review [5] and recommended in two additional reviews [6, 7]. Following the Medical Research Council’s framework for designing and evaluating complex interventions, we need to assess predefined progression criteria to reduce doubts about sample size, recruitment, and data collection in a future intervention study. Importantly, our study recognizes the moderate correlation between the tests; however, this observation does not overshadow the central focus on the Mindmore tablet’s effectiveness in assessing cognitive domains and do not diminish the potential of using tablet in the perioperative setting.

Our study illuminates that digital test batteries provides rapid access to results from validated test scores, consequently eliminating the time-consuming need to score participants manually. Self-administered digital tests increase the accessibility of cognitive screening and provide standardized execution without a test leaders’ influence, a high measure of reliability, and automated data collection [7, 44].

The preference expressed by several interviewed participants for the digital test regardless of their prior digital device experience, underscores the user-friendly nature of the tablet from Mindmore. Participants appreciated its clarity and understandability, and the availability of having staff close by, further contributed to a sense of security during the test sessions aligning with findings from previous research [45, 46].

Given the challenges presented by COVID-19 pandemic, the need for digital alternatives has proven crucial for health care delivery for vulnerable patient groups [47], and is an effective way of providing care and support to older adults [46]. Previous research has found that older adults face several challenges when using digital technologies, such as information overwhelm or difficult user interfaces; however, in our study, participants found the digital tablet easy and clear to use.

### Strengths and limitations

This study has some strengths—to our knowledge, no other study has compared the ISPOCD test battery with a digital counterpart or interviewed older adult participants about their experiences after undertaking digital cognitive tests.

There are also some limitations to this study. We modified the SUS scale [31], where we chose the questions most suitable for this digital test, meaning we did not use the initial method of calculating the score. Instead, we presented the results with means and SD. Moreover, the variability of our chosen tests differed more than expected from the ISPOCD battery. Opting for tests that strictly mirrored the ISPOCD might have produced different results. Our sample of fifty is a small, homogeneous, highly educated cohort of Swedish-speaking participants, which does not represent the entire population of older adults in Sweden. We assumed the cohort was cognitively healthy since they reported themselves to be so, but we cannot be sure, as we did not conduct a minor cognitive screening such as the Mini Mental State Examination [48] before the tests. Additionally, we lack information about multi-morbidities such as vascular disease or heart failure, which may impact cognitive performance.

## Conclusion

In conclusion, our study provides valuable insights into the shift from analog neurocognitive tests to digital tests in the field of perioperative cognitive assessment. Through our comparison of the analog ISPOCD test battery with the digital Mindmore test, we have identified several advantages associated with digital testing, including high usability, rapid test results, and participant preference. Notably, our study represents the first attempt to directly compare these two test batteries, making a contribution to the existing literature. These findings suggest that digital neurocognitive tests may offer a promising alternative to traditional analog tests in the field of perioperative neurocognitive disorders. However, further research is necessary to evaluate the feasibility of implementing the Mindmore test within the perioperative clinical setting. In summary, our study underscores the importance of ongoing research aimed at improving the accessibility of cognitive assessment for vulnerable patient populations.

## Data Availability

The data that supports the findings are available from the corresponding author at reasonable request.

## Abbreviations

BNT-15: Boston Naming Test-15
CERAD: Consortium to Establish a Registry for Alzheimer´s Disease
CST: Concept shifting task
dNCR: Delayed neurocognitive recovery
GDS-15: Geriatric depression scale-15
ISPOCD: International study of postoperative cognitive dysfunction
LDC: Letter digit coding test
MSCW: Mindmore Stroop color word test
PASAT: Paced Auditory Serial Addition Test
p-NCD: postoperative neurocognitive disorder
POCD: Postoperative cognitive dysfunction
RAVLT: Rey’s Auditory Visual Learning Test
SCW: Stroop color word test
SDPT: Symbols digit processing test
SUS: System usability scale
SwQoR: Swedish quality of recovery
TMT A&B: Trail Making Test A and B
VVLT: Visual Verbal Learning Test
